# Risk factors related to preventable infant mortality in Espirito Santo, Brazil

**DOI:** 10.1016/j.heliyon.2022.e12227

**Published:** 2022-12-26

**Authors:** Barbara Almeida Soares Dias, Katrini Guidolini Martinelli, Luiz Carlos de Abreu, Edson Theodoro dos Santos-Neto

**Affiliations:** a Federal University of Roraima, Boa Vista, RR, Brazil; b Graduate Program in Public Health. Federal University of Espirito Santo, Brazil; c School of Medicine, Masters of Science in Public Health, University of Limerick, Ireland

**Keywords:** Child health, Infant mortality, Cause of death, Analytical epidemiology

## Abstract

**Objective:**

To analyse the factors associated with preventable of infant mortality, in Espirito Santo, Brazil.

**Methods:**

Data were collected from records of notifiable of infant death of the Mortality Information System. A total of 5,089 infant deaths were classified as preventable and non-preventable according to the International Collaborative Effort on Infant Mortality (ICE) and the State System of Data Analysis Foundation (SEADE) methods. To investigate the factors associated with preventable of deaths, it was applied the logistic regression.

**Results:**

Approximately, 73% of the deaths were preventable according to the ICE, while 76% were preventable according to the SEADE method. Using to both methods, it was observed that preterm birth, postneonatal death and birth weight between 3000 and above 4,000 g represented higher chances for preventable infant deaths. Furthermore, the medical care was more likely to preventable infant death only for ICE method.

**Conclusions:**

The factors related to the quality of care offered in the prenatal, prepartum and childbirth periods were more relevant for the occurrence of preventable infant death. Accordingly, it is recommended to strengthen mother-child care to detect risk pregnancies during prenatal care, as well as a hierarchical, regionalized and integrated perinatal network.

## Highlights points

• Construction of algorithms for classification methods of preventable causes of infant death.
• Classification of causes of deaths as preventable and non-preventable using ICE and SEADE methods.
• Analysis of factors related to preventable infant death using the ICE and SEADE methods.
• The factors related to prenatal, prepartum and childbirth were more relevant to preventable infant mortality.

## 1 Introduction

Infant mortality (IM) is a major indicator for evaluating the living conditions of a population, and is also a factor that reveals the quality of health services [1, 2]. Several studies have been indicated a decline in the infant mortality rate in recent decades in Brazil, vis-à-vis new mother–child policies and strategies for promoting health and preventing disease [3, 4, 5].

It is evidenced that this decline was mainly reflected in the postneonatal component of IM, since these deaths are more susceptible to low-cost actions and use of more simplified technologies. On the other hand, for the neonatal component, there has been a slow decrease in recent years, resulting from factors related to the quality of healthcare during the prenatal, prepartum and childbirth periods, and to the newborn infant, in addition to biological and socioeconomic factors [6, 7]. Thus, it is understood that actions of prevention during the neonatal period are more complex, requiring interventions especially during the prenatal period and during the hospital stay, for example, in the care of the mother and the high-risk newborn.

However, the determinants for IM are clearly defined in the scientific literature, mostly considering preventable events. Nonetheless, the factors associated with preventability of infant mortality have been poorly described. The factors associated with preventable and non-preventable deaths are mainly related to the quality of care offered to pregnant women and newborns [8]. But also, the lack of a hierarchical network with regional reference centers for perinatal and neonatal care, such as Neonatal Intensive Care Units (NICUs), plus the persistence of important inequalities in the distribution of preventable deaths in Brazilian cities and towns, reinforce the challenges still present in our society, such as the family's low rate of participation in health services and the low number of health professionals in remote areas [9].

Health inequities, difficulties in accessing health services, poor living conditions, and lack of safe transportation of the newborn are determinants for the occurrence of infant mortality, mainly for deaths due to preventable causes. According to the World Health Organization (WHO), millions of children die annually from preventable causes and treatable diseases, despite the scientific knowledge and technologies available [10].

Coupled with this, in recent years, several authors have developed systems to classify the causes of death as preventable or non-preventable, such as: Taucher [11]*,* Wigglesworth [12], International Collaborative Effort on Infant Mortality (ICE) [13], State System of Data Analysis Foundation (SEADE) [14], and Brazilian List of Infant Death Preventability [15]. It is emphasized that these methods encompass, within their systems, the determinants for the occurrence of death.

The use of these preventable methods, as instruments to assist in the investigation of death, makes it possible to detect the factors that determine the cause thereof, and thus contribute to the development of actions geared toward populations at greater risk [8]. However, these interventions must be proposed taking into account the needs of different social groups, and according to regional differences.

Aiming to understand the relationship between factors involved in preventable infant deaths, the purpose of this study is to analyse the factors associated with preventable infant mortality according to the ICE [13] and SEADE [14] methods, in the state of Espirito Santo, Brazil.

## 2 Methods

An analytical descriptive study was carried out based on secondary data. In the period from 2006 to 2013, there were 5,316 deaths of children under one year of age in Espirito Santo, Brazil. Of this total, 227 cases were excluded because they presented birth weight less than 500 g and because birth occurred after a gestation period of less than 22 weeks.

The data were obtained through the records of notification of neonatal and infant death, scanned and provided by the Health Secretariat of Espirito Santo (SESA-ES). The period after 2006 was estimated due to the availability of the data in the standardized system in SESA-ES and the adequacy of this data to the practices in the Manual of the Committees for the Prevention of Infant and Fetal Death.

**Figure 1:**
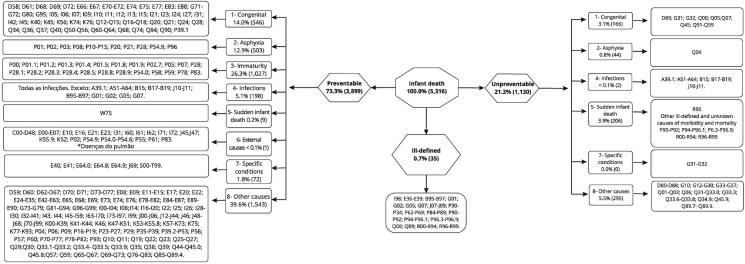
Application of the ICE method to the classification of infant deaths. Espírito Santo State, Brazil, 2006–2013. From: Dias et al. [16].

**Figure 2:**
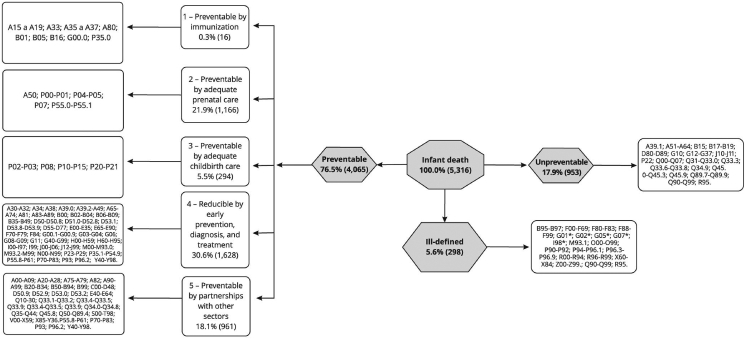
Application of the SEADE Foundation method to the classification of infant deaths. Espírito Santo State, Brazil. 2006–2013. From: Dias et al. [16].

The underlying cause of death, coded according to the 10^th^ Revision of the International Classification of Diseases (ICD-10), was classified as preventable or non-preventable to obtain the outcome, according to the following methods: ICE [13] and SEADE [14], because other methods had less capacity to discriminate preventable death [16]. For this, algorithms were built for ICE [13] and SEADE [14] preventable methods through the analysis of each cause of infant death recorded in the notification form (Figures 1 and 2). Thus, it was possible to classify the codes of each death according the preventable methods proposed.

The ICE [13] method, in its framework, considers eight groups of causes of death, namely: Congenital; Asphyxia; Immaturity; Infection; Sudden Infant Death (SIDS); External causes; specific conditions; and other causes (Figure 1). While, the SEADE [14] method proposes three groups of cause of death: preventable causes, non-preventable causes and poorly-defined causes. The group of preventable causes is subdivided into: Causes reducible by immunoprevention; by appropriate control during pregnancy; by appropriate attention to childbirth; by actions of prevention, diagnosis, and early treatment; and through partnerships with other sectors. However, for this study, only the dichotomous qualification between preventable deaths and non-preventable deaths was considered (Figure 2). More details about the ICE [13] and SEADE [14] methods can be consulted in Dias et al [16].

The variables of interest were extracted from the Mortality Information System (SIM) maintained by Brazil's Ministry of Health. It is worth noting that Espirito Santo has good coverage (equal to or greater than 90%) of the SIM [17]. The factors used for the study were: year in which death occurred (2006–2013); age at death (neonatal/postneonatal); assigned sex (men/women); self-reported skin color (white/black/brown); region where death occurred (north/central/metropolitan/south); place where death occurred (hospital/non-hospital); child healthcare (public/private); mother's age (9–14/15-19/20–34/≥35 years); mother's occupation (work at home/work outside/student); number of living children and number of deceased children from the same mother (none/one/two or more); type of pregnancy (single/double or more); gestational age estimated preferentially by last menstrual period (LMP) or ultrasound and physical examination when LMP is unknown [18] (<37 weeks/37–41 weeks/≥42 weeks); type of delivery (vaginal/cesarean); death in relation to childbirth (intrapartum/after); birth weight (<2,500 g/2,500 g to 3,999 g/above 4,000 g); medical care; and surgery (yes/no).

In the statistical analysis, the chi-square test was initially applied to verify the differences in proportions between the independent variables and preventable mortality, according to the preventable methods ICE and SEADE.

Next, binary logistic regressions analyses were used to evaluate the effects of the independent variables on the outcome, calculating the crude odds ratios (OR). Then, all independent variables with p-value less than 20% were included in the multiple logistic regressions. We performed multiple logistic regressions for each preventable method, in order to adjust the results for potential confounders. Additionally, we tested the effects for interaction before the final analysis in all regressions, and when they were present, they were maintained in the final analyses. The models were manually tested and the one whose pseudo-R2 statistic (Cox & Snell e Nagelkerke) value was closest to 1 and the Hosmer-Lemeshow test showed p > 0.05 was chosen with the best fit. For statistical treatment, version 21 of statistical package for the social sciences (SPSS) software was used, considering the 95% confidence interval (95% IC).

This study was reviewed by the Research Ethics Committee (CEP) of the Health Sciences Center, Federal University of Espírito Santo, under the report number 999.562 and Certificate of Presentation for Ethical Review (CAAE) number 42695015.7.0000.5060. In addition, the Term of Commitment for the use of institutional data approved by SESA-ES was signed on October 10, 2014.

## 3 Results

A total of 5,089 deaths of children less than one year of age reported in Espirito Santo from 2006 to 2013 were analyzed. Among the 5,089 deaths of children less than one year old, the ICE [13] classified a total of 4,805 (94.4%) deaths, of which 72.9% as preventable and 21.5% non-preventable, since 35 (0.7%) deaths occurred due to poorly-defined causes and 249 (4.9%) deaths remained unclassified. On the other hand, the SEADE [14] classified 4,795 (94.2%) deaths, of which 76.1% as preventable and 18.1% non-preventable, since 294 (5.8%) deaths occurred due to poorly-defined causes.

Table1 describes the characteristics between preventable and non-preventable infant deaths according ICE [13] and SEADE [14] methods. Note that in all characteristics, there was a higher occurrence of preventable infant deaths in both methods, highlighting the age at death, place of death, and gestational age.

**Table 1:** Characteristics of preventable and non-preventable infant deaths according ICE [13] and SEADE [14] methods, Espirito Santo, 2006–2013.

	ICE			SEADE		
	Non-preventable (%)	Preventable (%)	p-value	Non-preventable (%)	Preventable (%)	p-value
Year of death						
2006–2007	307 (28.0)	1066 (28.7)	0.010	236 (25.6)	1101 (28.4)	0.013
2008–2009	290 (26.5)	949 (25.6)		246 (26.7)	1009 (26.0)	
2010–2011	290 (26.5)	844 (22.7)		249 (27.0)	871 (22.5)	
2012–2013	208 (19.0)	851 (22.9)		190 (20.6)	893 (23.1)	
Age at death						
Neonatal	831 (75.9)	2587 (69.7)	<0.001	751 (81.5)	2620 (67.6)	<0.001
Postneonatal	264 (24.1)	1123 (30.3)		170 (18.5)	1254 (32.4)	
Assigned sex						
Men	574 (53.3)	2093 (57.0)	0.290	486 (53.8)	2200 (57.4)	0.054
Women	503 (46.7)	1578 (43.0)		417 (46.2)	1636 (42.6)	
Self-reported skin color						
White	382 (43.7)	1271 (43.1)	0.800	351 (47.6)	1322 (42.9)	0.042
Black	25 (2.9)	97 (3.3)		17 (2.3)	101 (3.3)	
Brown	467 (53.4)	1578 (53.6)		369 (50.1)	1656 (53.8)	
Region where death occurred						
North	148 (13.5)	473 (12.8)	0.178	117 (12.7)	503 (13.0)	0.782
Central	159 (14.5)	566 (15.3)		140 (15.2)	584 (15.1)	
Metropolitan	611 (55.9)	1974 (53.3)		502 (54.6)	2054 (53.1)	
South	176 (16.1)	693 (18.7)		161 (17.5)	729 (18.8)	
Place where death occurred						
Hospital	992 (90.8)	3490 (94.3)	<0.001	889 (96.8)	3545 (91.7)	<0.001
Non-Hospital	100 (9.2)	212 (5.7)		29 (3.2)	319 (8.3)	
Child healthcare						
Public	856 (84.7)	3056 (86.3)	0.187	757 (84.2)	3110 (86.4)	0.088
Private	155 (15.3)	485 (13.7)		142 (15.8)	489 (13.6)	
Mother's age						
9–14 years	10 (1.2)	34 (1.2)	0.935	9 (1.3)	36 (1.2)	0.761
15–19 years	177 (21.2)	574 (20.2)		149 (20.8)	591 (20.2)	
20–34 years	544 (65.3)	1877 (66.1)		462 (64.5)	1939 (66.4)	
≥35 years	102 (12.2)	354 (12.5)		96 (13.4)	354 (12.1)	
Mother's occupation						
Work at home	367 (55.0)	1330 (58.4)	0.289	304 (53.7)	1374 (58.7)	0.100
Work outside	249 (37.3)	796 (34.9)		219 (38.7)	812 (34.7)	
Student	51 (7.6)	153 (6.7)		43 (7.6)	156 (6.7)	
Number of living children						
None	134 (18.4)	492 (19.6)	0.732	117 (18.9)	498 (19.2)	0.974
One	280 (38.5)	975 (38.8)		243 (39.3)	1006 (38.9)	
Two or more	313 (43.1)	1049 (41.7)		258 (41.7)	1084 (41.9)	
Number of deceased children from the same mother						
None	519 (83.8)	1697 (79.5)	0.029	438 (83.4)	1749 (79.5)	0.025
One	81 (13.1)	328 (15.4)		73 (13.9)	335 (15.2)	
Two or more	19 (3.1)	109 (5.1)		14 (2.7)	116 (5.3)	
Type of pregnancy						
Single	858 (92.5)	2814 (90.1)	0.031	738 (92.0)	2888 (90.2)	0.114
Double or more	70 (7.5)	309 (9.9)		64 (8.0)	314 (9.8)	
Gestation age						
<37 weeks	571 (64.1)	1903 (63.4)	0.317	517 (66.5)	1919 (62.5)	0.011
37–41 weeks	312 (35.1)	1057 (35.2)		257 (33.1)	1108 (36.0)	
≥42 weeks	7 (0.8)	43 (1.4)		3 (0.4)	45 (1.5)	
Type of delivery						
Vaginal	389 (42.9)	1469 (47.4)	0.015	306 (39.0)	1516 (47.8)	<0.001
Cesarean	518 (57.1)	1627 (52.6)		479 (61.0)	1655 (52.2)	
Death in relation to childbirth						
Intrapartum	0	0	-	0	0	
After	971 (100)	3280 (100)		828 (100)	3368 (100)	
Birth weight						
<2,500 g	615 (69.3)	1925 (63.8)	0.007	558 (72.1)	1934 (62.8)	<0.001
2,500–3,999 g	257 (28.9)	1009 (33.4)		203 (26.2)	1063 (34.5)	
≥4,000 g	16 (1.8)	83 (2.8)		13 (1.7)	85 (2.8)	
Medical care						
Yes	730 (92.5)	2631 (96.0)	<0.001	656 (98.2)	2672 (93.7)	<0.001
No	59 (7.5)	107 (3.9)		12 (1.8)	180 (6.3)	
Surgery						
Yes	46 (9.0)	226 (13.6)	0.006	42 (10.3)	236 (13.5)	0.086
No	464 (91.0)	1437 (86.4)		366 (89.7)	1518 (86.5)	

The variables such as year of death, age at death, place of death, number of deceased children from the same mother, gestational age, type of delivery, birth weight, medical care during childbirth and surgical procedures performed on the child presented significance <20% for both preventability methods. In addition to these, the variable type of pregnancy was also found for the ICE [13], and for the SEADE [14] the variables assigned sex, establishment where death occurred and confirmation of diagnosis by autopsy (Table 1).

According to ICE [13] method, it was observed that postneonatal deaths had a chance around 50% higher that the death would be considered as preventable than deaths occurring in the neonatal period (adjusted OR = 1.53; 95% CI = 1.23–1.91) (Table 2). For the SEADE [14], the chance was 90% higher (adjusted OR = 1.90; 95% CI = 1.49–2.44) (Table 3).

**Table 2:** Characteristics associated with preventable infant deaths according ICE [13] method. Espirito Santo, Brazil. 2006–2013.

	OR*	95% IC	Adjusted OR**	95% IC
Age at death				
Neonatal Postneonatal	1.00 1.37	- 1.17–1.60	1.00 1.53	- 1.23–1.91
Place where death occurred				
Non-hospital	1.00	-	-	-
Hospital	1.67	1.30–2.13	-	-
Number of deceased children from the same mother				
None	1.00	-	-	-
One	1.24	0.95–1.61	-	-
Two or more	1.76	1.07–2.88	-	-
Type of pregnancy				
Single	1.00	-	1.00	-
Double or more	1.35	1.03–1.76	1.43	1.03–1.96
Type of delivery				
Cesarean	1.00	-	-	-
Vaginal	1.20	1.04–1.40	-	-
Gestational age				
<37 weeks	0.98	0.84–1.15	0.68	0.45–1.03
37–41 weeks	1.00	-	1.00	-
≥42 weeks	1.81	0.81–4.07	2.75	0.63–11.89
Birth weight				
<2,500 g	0.80	0.68–0.94	0.44	0.31–0.63
2,500–3,999 g	1.00	-	1.00	-
>4,000 g	1.32	0.76–2.30	1.16	0.59–2.29
Gestational age * Birth weight	-	-	2.67	1.58–4.51
Medical care				
No	1.00	-	1.00	-
Yes	1.99	1.43–2.76	2.70	1.79–4.06
Surgery				
No	1.00	-	-	-
Yes	1.59	1.14–2.21	-	-

95%CI: 95% confidence interval; OR: odds ratio.

Hosmer-Lemeshow test: p-value = 0.179.

* All variables were tested with p-value <0.20. However, only ones considered in the table where the ones tested according to the final model.

** OR adjusted by the variables that continued in the final model and year of death.

**Table 3:** Characteristics associated with preventable infant deaths according SEADE [14] method. Espirito Santo, Brazil. 2006–2013.

	OR*	95% IC	Adjusted OR**	95% IC
Age at death				
Neonatal	1.00	-	1.00	
Postneonatal	2.11	1.77–2.53	1.90	1.49–2.44
Assigned sex				
Men	1.00	-	-	-
Women	0.87	0.75–1.00	-	-
Self-reported skin color				
White	1.00	-	-	-
Black	1.58	0.93–2.67	-	-
Brown	1.19	1.01–1.40	-	-
Place where death occurred				
Non-hospital	1.00	-	-	-
Hospital	0.36	0.25–0.53	-	-
Child healthcare				
Private	1.00	-	-	-
Public	1.19	0.97–1.46	-	-
Number of deceased children from the same mother				
None	1.00	-	-	-
One	1.15	0.87–1.51	-	-
Two or more	2.08	1.18–3.64	-	-
Type of delivery				
Cesarean	1.00	-	-	-
Vaginal	1.43	1.22–1.68	-	-
Gestational age				
<37 weeks	0.86	0.73–1.02	0.67	0.43–1.04
37–41 weeks	1.00	-	1.00	-
≥42 weeks	3.48	1.07–11.28	‡	‡
Birth weight				
<2,500 g	0.66	0.55–0.79	0.39	0.27–0.56
2,500–3,999 g	1.00	-	1.00	
≥4,000 g	1.25	0.68–2.28	1.16	0.56–2.42
Gestational age * Birth weight	-	-	3.05	1.76–5.30
Medical care				
No	1.00	-	1.00	-
Yes	0.27	0.15–0.49	0.51	0.26–1.00
Surgery				
No	1.00	-	-	-
Yes	1.36	0.96–1.92	-	-

95%CI: 95% confidence interval; OR: odds ratio.

Hosmer-Lemeshow test: p-value = 0.241.

* All variables were tested with p-value <0.20. However, only ones considered in the table where the ones tested according to the final model.

** OR adjusted by the variables that continued in the final model and year of death.

‡ Due to a small numbers of cases, the adjusted ORs could not be calculated.

In relation to type of pregnancy, it was observed that the multiple pregnancy had a chance 43% higher be considered preventable deaths (adjusted OR = 1.43; 95%CI = 1.03–1.96) considering ICE [13] method (Table 2).

The birth weight was shown to be a relevant variable for determining preventable infant death in both methods, presenting a gradient in the estimates (increase in OR) as birth weight increases. The birth weight <2,500 g had 56% lower chance to be considered preventable death for the ICE [13] method (Table 2) and 61% lower chance to preventable death by SEADE [14] method (Table 3).

Considering medical care at the time of childbirth, there was observed 2.70 times the chance of the death was preventable, compared to children who died without medical intervention in the ICE [13] method (Table 2).

## 4 Discussion

In this study, it was observed that the ICE [13] method classified a higher number of deaths as preventable and non-preventable than the SEADE [14] method. This can be explained by differences in their methodological constructs, since the SEADE [14] method encompasses the causes of deaths preventable by actions of immunoprevention, unlike the ICE [13] method, which does not consider such actions. However, the preventability criteria adopted by the various preventability methods are not definitive and vary according to the health services available, technological advances and social determinants that exist in each country [19].

The results indicated that the factors that remained in the final model were mainly those relating to the child's biological and birth conditions. Studies have shown that qualified healthcare for mother and child represents a major factor for the reduction of infant mortality [8, 20].

The postneonatal death was associated with increased odds of preventable death, similarly to the case-control study carried out in Rio Grande do Sul, Brazil [8]. The causes of deaths in the postneonatal period are related mainly to infectious and parasitic diseases, i.e. they are associated with the environment in which the newborn is inserted. These factors are potentially preventable by public health measures, including immunization campaigns, antibiotic therapy, oral rehydration [21, 22], and breastfeeding in the first year of life, which can be the most feasible strategy for reducing postneonatal deaths [5]. Although these actions have an impact on reducing the postneonatal mortality rate in recent years, the study of the causes of these deaths is still relevant, as there are a high number of postneonatal deaths from preventable causes [23, 24].

Regarding of type of pregnancy, we observed that multiple pregnancy showed a greater odds of preventable death only for the ICE [13] method. It is recognized that multiple pregnancy presents greater risks for infant mortality, due to fetal growth restriction [25, 26, 27]. However, these deaths can be considered preventable, for the most part, provided that pregnant women have timely access to health services, as well as adequate and qualified prenatal care [25].

Although not significant, we observed that preterm birth had a lower chance of preventable death. Studies indicate a strong association between children born during a gestational period of less than 37 weeks and infant mortality [8, 28]. Factors related to social vulnerability and lifestyle strongly influence the occurrence of preterm births [29, 30], characterizing factors that can lead to infant death and future morbidities, and originate from causes considered as preventable.

At present, data shown that low birth weight was associated with lower odds of preventable infant death, which was consistent with other studies [8, 31]. A previous study carried out in Brazil, Lansky et al. [7] show that preterm infants with an extremely low birth weight had 200 to 300 times greater odds of dying in the neonatal period compared to full-term infants that were born weighing more than 2,500 g. However, low birth weight is strongly associated with both neonatal and postneonatal mortality [7, 8, 23].

Furthermore, medical care was associated with a 2.7 fold adjusted increase in preventable infant death, considering only ICE [13] method. The quality of medical care is directly related to infant mortality, as the research by Lansky et al. [7] identified that the absence of good practices during labor poses a five-fold risk for the occurrence of neonatal mortality and a nearly three-fold risk during childbirth. The study also found that non-recommended practices were frequently performed, while necessary practices, such as a partogram were hardly used. This reinforces the importance of good practices in labor and delivery for the effectiveness of the quality of mother-child care, since it reduces the stressful situations to which the expectant women are exposed and, consequently, the reduction of infant mortality due to preventable causes.

According to the methods, the factors relating to the preventability of infant death also come mainly from prenatal care. The adequate prenatal care is essential for the monitoring the health of the mother and child, providing for a safe gestation by incorporating nurturing conduct and allowing the detection and early intervention of risky pregnancies as well as possible abnormalities, especially those considered preventable. However, studies have shown that the use of health services are a result of situations such as social inequality and service availability, that is, white women from privileged socioeconomic groups living in more developed regions and with higher levels of schooling, presents more odds to adequate prenatal care [5, 32]. So, the inefficiency of this service, due to failures in monitoring pregnant women [7, 8, 33, 34], contributes to the increase in infant mortality, specially, from preventable causes.

The present study did not show statistically significant differences between sociodemographic factors and preventable infant deaths, even though greater risks of these deaths are recognized in regions of great social vulnerability. An ecological study carried out in Espírito Santo, Brazil, identified that furthest areas from the urban center and with greatest lack of services had the highest rates of preventable infant mortality [35], pointing to a difficulty in accessing health services due to inequality in resources and investments.

In addition, our results showed higher proportions of preventable infant deaths in the public health sector, which disproportionately meet the high level of women of lower socioeconomic status. In addition, we observed greater proportions of infant deaths among black and brown mothers, who have more difficulties in accessing prenatal, childbirth and post-neonatal care due to the barriers that institutional racism imposes. A nationwide population-based retrospective cohort study, analyzing the inequalities in childhood mortality rates by maternal race and skin color, revealed higher odds of mortality among children younger than 5 years of brown or mixed race and black mothers, compared with children of white mothers, being the odds even higher in the post-neonatal period [36].

This reinforces that there are still challenges to be faced for the reduction of infant deaths from preventable causes, even with the increased coverage of health services and improvements in the quality of mother-child assistance in recent years. Therefore, this study highlights the importance of the applicability of these methods to evaluate preventable deaths to assess the quality of healthcare, since it would be possible to identify the causes of the deaths and, consequently, to propose health strategies to prevent them. Furthermore, the use of preventable death indicators has been a useful tool for monitoring the impact of the health sector on the risk of death in populations.

On the other hand, the study was limited to the variables contained on the Fetal and Neonatal Death Notification Form, and it was not possible to use variables related to clinical conditions of death, such as the Apgar score at one and five minutes, described by authors as a factor related to infant mortality [7, 28, 37, 38]. Additionally, a major problem in the analysis of infant deaths is the availability of reliable information about registration of the death [9] and validity of underlying cause of death, since the database is secondary and results from the death certificate is issued by the health service. In this study, we observed the absence of some data, and it was not possible to include data on prenatal care, since this variable is not available in the SIM; but this does not nullify the results achieved, vis-à-vis the size of the sample used.

The use of infant death classification methods was an advance for preventable death analyzes in Brazil. From this, the results of this study showed high preventable infant deaths in the Espírito Santo, being associated with biological factors, care during the prenatal, prepartum period, childbirth, and to the newborn child. In this regard, there is a need to strengthen a network of hierarchical, regionalized and integrated perinatal care, with massive investment in training and education of health professionals, since children are dying due to lack of proper care. Also, this study emphasizes the importance of strengthening the Fetal and Infant Death Prevention Committees as instruments for research into the causes of death, in order to intensify the actions aimed at reducing deaths from preventable causes.

## Declarations

### Author Contribution statement

Barbara Almeida Soares Dias, Katrini Guidolini Martinelli, Luiz Carlos de Abreu and Edson Theodoro dos Santos-Neto: Conceived and designed the experiments; Performed the experiments; Analyzed and interpreted the data; Contributed reagents, materials, analysis tools or data; Wrote the paper.

### Funding statement

This research did not receive any specific grant from funding agencies in the public, commercial, or not-for-profit sectors.

### Data availability statement

Data included in article/supp. material/referenced in article.

### Declaration of interests statement

The authors declare no conflict of interest.

### Additional information

No additional information is available for this paper.
